# Machine Learning CT-Based Automatic Nodal Segmentation and PET Semi-Quantification of Intraoperative ^68^Ga-PSMA-11 PET/CT Images in High-Risk Prostate Cancer: A Pilot Study

**DOI:** 10.3390/diagnostics13183013

**Published:** 2023-09-21

**Authors:** Guido Rovera, Serena Grimaldi, Marco Oderda, Monica Finessi, Valentina Giannini, Roberto Passera, Paolo Gontero, Désirée Deandreis

**Affiliations:** 1 Nuclear Medicine, Department of Medical Sciences, AOU Città della Salute e della Scienza di Torino, University of Turin, 10126 Turin, Italy; guido.rovera@unito.it (G.R.);; 2 Urology Unit, Department of Surgical Sciences, AOU Città della Salute e della Scienza di Torino, Molinette Hospital, University of Turin, 10126 Turin, Italy; 3 Department of Surgical Sciences, University of Turin, 10126 Turin, Italy; 4 Nuclear Medicine Division, Gustave Roussy, 94805 Villejuif, France

**Keywords:** PET/CT, PET/CT specimen images, ^68^Ga-PSMA-11, prostate cancer, automatic segmentation, machine learning

## Abstract

High-resolution intraoperative PET/CT specimen imaging, coupled with prostate-specific membrane antigen (PSMA) molecular targeting, holds great potential for the rapid ex vivo identification of disease localizations in high-risk prostate cancer patients undergoing surgery. However, the accurate analysis of radiotracer uptake would require time-consuming manual volumetric segmentation of 3D images. The aim of this study was to test the feasibility of using machine learning to perform automatic nodal segmentation of intraoperative ^68^Ga-PSMA-11 PET/CT specimen images. Six (*n* = 6) lymph-nodal specimens were imaged in the operating room after an e.v. injection of 2.1 MBq/kg of ^68^Ga-PSMA-11. A machine learning-based approach for automatic lymph-nodal segmentation was developed using only open-source Python libraries (Scikit-learn, SciPy, Scikit-image). The implementation of a k-means clustering algorithm (*n* = 3 clusters) allowed to identify lymph-nodal structures by leveraging differences in tissue density. Refinement of the segmentation masks was performed using morphological operations and 2D/3D-features filtering. Compared to manual segmentation (ITK-SNAP v4.0.1), the automatic segmentation model showed promising results in terms of weighted average precision (97–99%), recall (68–81%), Dice coefficient (80–88%) and Jaccard index (67–79%). Finally, the ML-based segmentation masks allowed to automatically compute semi-quantitative PET metrics (i.e., SUVmax), thus holding promise for facilitating the semi-quantitative analysis of PET/CT images in the operating room.

## 1. Introduction

Molecular imaging with prostate-specific membrane antigen (PSMA) positron emission tomography/computed tomography (PET/CT) has emerged as one of the leading diagnostic procedures to investigate prostate cancer (PCa) patients, due to its high diagnostic accuracy in detecting disease localizations in biochemically recurrent PCa [1,2,3,4,5,6,7,8,9], as well as its promising results in the setting of primary staging [10,11,12,13,14]. Indeed, PSMA PET/CT has shown superior performance compared to other molecular imaging techniques, such as choline-PET [7] and fluciclovine-PET [8], to correctly locate the site of disease recurrence. Thanks to the improved restaging accuracy and enhanced target delineation, PSMA-PET holds potential to significantly impact the management of recurrent prostate cancer, allowing for more effective imaging-guided treatments [2,9]. Beyond the setting of biochemical recurrence, in recent years, an increasing number of studies have also evaluated the diagnostic performance of PSMA PET for the primary staging of PCa, using either MRI- or CT-hybrid scanners [12,13,15,16]. In the proPSMA trial by Hofman et al. [10], PSMA PET/CT showed greater accuracy (92% vs. 65%), sensitivity (85% vs. 38%) and specificity (98% vs. 91%), compared to conventional imaging (i.e., CT and bone scintigraphy), for identifying pelvic nodal or distant-metastatic localizations in high-risk localized PCa patients. Furthermore, PSMA PET/CT was associated with a higher rate of management changes (28% vs. 15%), less equivocal findings (7% vs. 23%), and a lower radiation exposure (8.4 mSv vs. 19.2 mSv).

Together with the development of novel radiopharmaceuticals, current technological improvements in PET/CT imaging are revolutionizing the field of molecular imaging, leading to enhanced diagnostic capabilities. Recently, a new mobile PET/CT specimen imager (AURA 10 specimen PET/CT imager, XEOS Medical NV, Gent, Belgium) has become available for specific use in the operating room, providing surgeons and imaging specialists the ability to perform intraoperative molecular imaging on resected specimens. This new device generates high-resolution 3D PET/CT specimen images within a few minutes from the excision and with a near five-fold increase in spatial resolution, thus supporting the assessment of the resection margin’s status, facilitating the detection of nodal metastases and holding promise for potentially guiding surgical procedures in the future. Intraoperative PET/CT specimen imaging has shown promising results in multiple oncological settings, including ^18^F-FDG avid breast cancer [17], head and neck cancer [18], pancreatic adenocarcinoma [19], as well as SSTR-expressing neuroendocrine tumors and PSMA-expressing prostate cancer [20,21].

The new possibilities offered by intraoperative PET/CT imaging, coupled with the specificity of PSMA molecular targeting, hold great potential for improving the staging accuracy and guiding robot-assisted radical prostatectomy (RARP) and pelvic lymph node dissection (PLND) in prostate cancer patients. However, in order to accurately analyze the radiotracer uptake in the histological specimens, time-consuming manual volumetric segmentation of 3D tomographic images would be necessary. Currently, there is great interest in the use of artificial intelligence and machine learning for automatic segmentation of ^18^F-FDG, ^68^Ga-labeled somatostatin analogs and PSMA whole-body PET/CT images [22,23,24,25,26,27,28,29,30]. However, no previous studies have documented the application of similar approaches in the context of intraoperative PET/CT specimen images. Therefore, the aim of this pilot study was to test the feasibility of using a machine learning algorithm to perform automatic nodal segmentation of intraoperative ^68^Ga-PSMA-11 PET/CT specimen images in prostate cancer patients.

## 2. Materials and Methods

A retrospective analysis was conducted on 3D high-resolution ^68^Ga-PSMA-11 PET/CT images of 6 surgical specimens obtained from a high-risk prostate cancer patient (PSA 16 ng/mL, Gleason Score 4 + 5 at biopsy, positive DRE, left extracapsular extension with possible infiltration of left seminal vesicle at MRI) undergoing robot-assisted radical prostatectomy (RARP) and pelvic lymph node dissection (PLND) with intraoperative use of the PET/CT specimen imager (AURA 10, XEOS Medical NV, Belgium). In accordance with the European Association of Urology (EAU) guidelines, the patient had previously undergone preoperative staging for distant metastases with computed tomography (CT) and bone scintigraphy (both negative), and was then referred for surgical treatment (RARP + PLND). Histopathological analysis was performed on the resected specimens. The details of the procedure are presented below. 

### 2.1. Radiopharmaceutical Synthesis and Surgical Procedure

The ^68^Ga-PSMA-11 was synthesized in the radiochemistry laboratory of the Division of Nuclear Medicine of the AOU Città della Salute e della Scienza, University of Turin, following established procedure guidelines [31] as previously documented [32]. Specifically, Gallium-68 was produced with a ^68^Ge/^68^Ga generator (ITM Isotope Technologies Munich SE, Garching bei München, Germany). ^68^Ga-PSMA-HBEDCC(Glu-NH-CO-NH-Lys-(Ahx)-[[68Ga]Ga(N,N′bis-[2-hydroxy-5-(carboxyethyl)benzyl]ethylenediamineN,N′-diacetic-acid]) (^68^Ga-PSMA-11) was prepared with a process comparable to the one described by Eder et al. [33], and transferred to cassette-based automated synthesis module (Module miniAllinOne, Trasis S.A, Ans, Belgium). The entire procedure was performed in adherence to the Good Manufacturing Practices (GMP), and the resulting solution (final product) was subjected to standard quality controls.

Prior to the procedure, signed informed consent was obtained from the subject. In the operating room, a dose of 157 MBq (2.1 MBq/kg) of ^68^Ga-PSMA-11 was administered intravenously to the patient during trocar placement. The surgery was performed with the Da Vinci Xi robot. Lymph nodes were extracted through the 12 mm assistant trocar and promptly examined using the specimen imager. The prostate was then removed through a Pfannestiel incision while maintaining CO_2_ insufflation, and scanned to assess for positive surgical margins (PSM) before proceeding with the urethra-vesical anastomosis.

### 2.2. Intraoperative PET/CT Imaging

Following excision in the operating room, the surgical specimens were placed in a dedicated specimen container and PET/CT images were acquired using the intraoperative scanner (AURA 10 specimen PET/CT imager, XEOS Medical NV, Belgium). On average, the time required by the PET/CT device to complete the scanning process was 12 min. CT images were reconstructed using the image space reconstruction algorithm at 100 µm voxel size. PET images were reconstructed using 20 iterations of the ordered subset expectation maximization (OSEM) algorithm at 400 µm voxel size. 

### 2.3. PET/CT Image Analysis: Automatic and Manual Segmentation

The specimen PET/CT images were processed using only open-source Python libraries or softwares. 

“NiBabel” was used to load the CT scan data extracted from the PET/CT images, while the “Sklearn” library was used to process the resulting “NumPy” arrays and identify nodal structures through a machine learning clustering algorithm (k-means clustering). Morphological operations (e.g., erosion and dilation) were applied to the clustered images using the “scipy.ndimage” and “skimage.morphology” libraries. Volumetric manual segmentation of nodal structures was performed with the ITK-SNAP software (v 4.0.1) using the polygon tool and considered as reference standard. Manual segmentation was performed by a nuclear medicine specialist with experience in diagnostic hybrid imaging and PSMA-PET/CT interpretation. CT-based manual contouring of nodal structures on all image slices required up to 15 min per lymph node.

The “sklearn.metrics” library was used to evaluate the accuracy of the machine-learning based 3D automatic segmentation compared to the manual segmentation by means of the following metrics:

- Accuracy: (TP + TN)/(TP + TN + FP + FN)
- Precision (Positive predictive value): TP/(TP + FP)
- Recall (Sensitivity): TP/(TP + FN)
- Dice coefficient (F1 Score): 2TP/(2TP + FP + FN)
- Jaccard index (Intersection-Over-Union)

where TP, TN, FP and FN represent true positives, true negatives, false positives and false negatives, respectively. The above metrics—as a measure of segmentation performance—represent the likelihood for a lymph node voxel to be correctly classified as belonging to a nodal structure; on the contrary, they do not evaluate the detection rate of pathological lymph nodes. Micro, macro, and weighted averages of the segmentation metrics were also provided; micro averages estimate the overall accuracy of the model across all classes; macro averages consider classes independently giving them equal importance; and weighted averages account for class imbalance.

Finally, the CT-based segmentation masks were applied to the PET images in order to automatically compute semi-quantitative measures of tracer distribution such as the maximum standardized uptake value (SUVmax) and the target-to-background ratio (TBR).

## 3. Results

**Figure 1:**
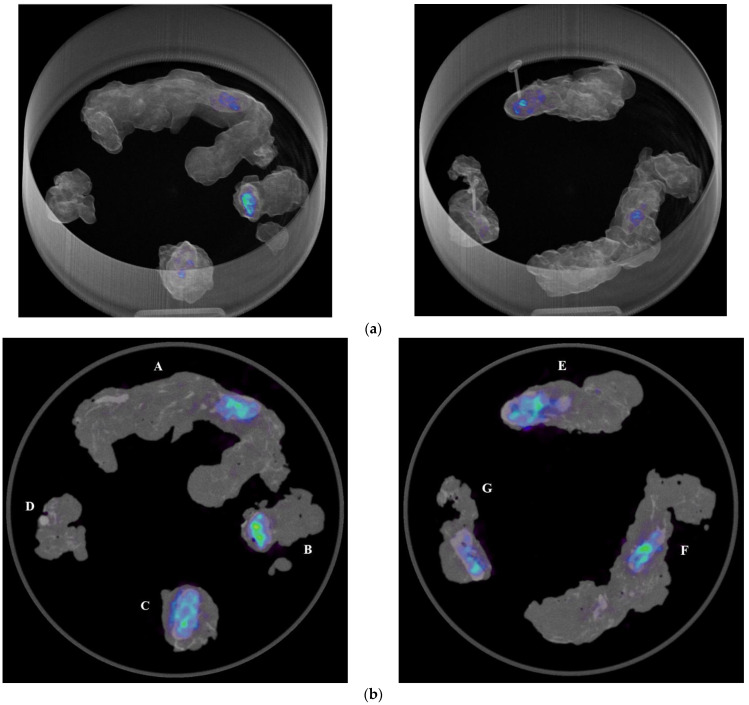
(**a**) Volumetric rendering and axial view (**b**) of ^68^Ga-PSMA-11 PET/CT specimen images of the pelvic lymph nodes of a high-risk prostate cancer patient undergoing robot-assisted radical prostatectomy (RARP) and pelvic lymph node dissection (PLND). A, B: left obturator, C: left proximal external iliac, D: left distal external iliac, E: right distal external iliac, F: right obturator, G: right proximal external iliac.

In the operating room, the acquisition of the specimens ^68^Ga-PSMA-11 PET/CT images with the intraoperative PET/CT scanner proved to be safe and feasible. The median time between radiotracer injection and specimen PET/CT imaging of the pelvic lymph nodes was 125 min. On average, the time required to complete the scanning process of a specimen case was 12 min. Figure 1a,b show the volume rendering of the PET/CT acquisitions, as well as two sample axial slices denoting mild tracer uptake in nodal structures.

The automatic segmentation of nodal structures was performed using only open-source Python libraries, such as NiBabel, Scikit-learn, SciPy and Scikit-image. The intraoperative PET/CT images proved to be a suitable setting for the application of automated segmentation based on machine learning due to the simplified anatomical context of the surgical specimens. The main steps of the segmentation model are described below.

**Figure 2:**
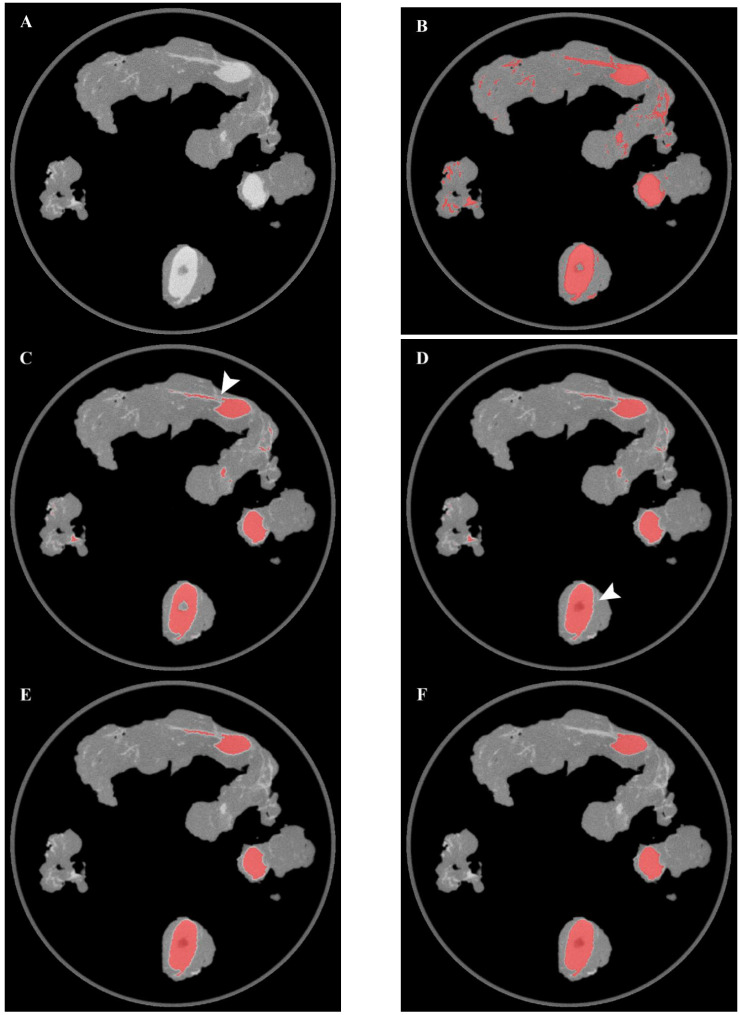
Overview of the results of the main segmentation steps on a sample axial image. (**A**) original CT image, (**B**) K-means clustering, (**C**) Erosion, (**D**) Filling, (**E**) 2D noise removal, (**F**) 3D noise removal. The erosion process allowed to detach two wrongly connected objects (arrow in panel (**C**)), while the hole-filling operation allowed to include the lymph node hilum in the segmented area (arrow in panel (**D**)).

First, leveraging the difference in tissue density between the lymph nodes and the surrounding structures, a k-means clustering algorithm (*n* = 3 clusters) was applied to one-dimensional CT attenuation data to identify nodal structures. The model was initialized with three clusters, thus allowing to discriminate between air, fat tissue, and lymph nodes, plus adjacent vessels/fibrotic tissue. Considering the one-dimensional data and the clusters number, the use of the default random starting points did not affect the results. The cluster corresponding to the fat tissue was then removed by assigning to its elements the same value of the background (air), thus creating a binary two-level mask of the nodal cluster. The specimen case was also removed from the images using a filter based on geometric distance in order to prevent background noise due to misclassification errors. Then, morphological operations of erosion and hole-filling were performed. The erosion process allowed not only to reduce image noise but also to detach wrongly connected objects such as vessels or fibrous tissues located in close proximity to a nodal structure. On the other hand, the filling operations also extended the segmented areas to regions with lower densities—such as the lymph node hilum—which would otherwise be excluded from clustering. Examples of the results of the morphological operations (erosion/filling) are presented in Figure 2. Noise removal was then performed on 2D slices by discarding features with a low number of elements. Then, morphological dilation was applied to compensate for the previous erosion process and also to join broken parts of the same elements. Finally, further noise removal was performed on the 3D image by discarding features with a lower volume. An overview of the results of the main segmentation steps on a sample axial image is shown in Figure 2. 

**Figure 3:**
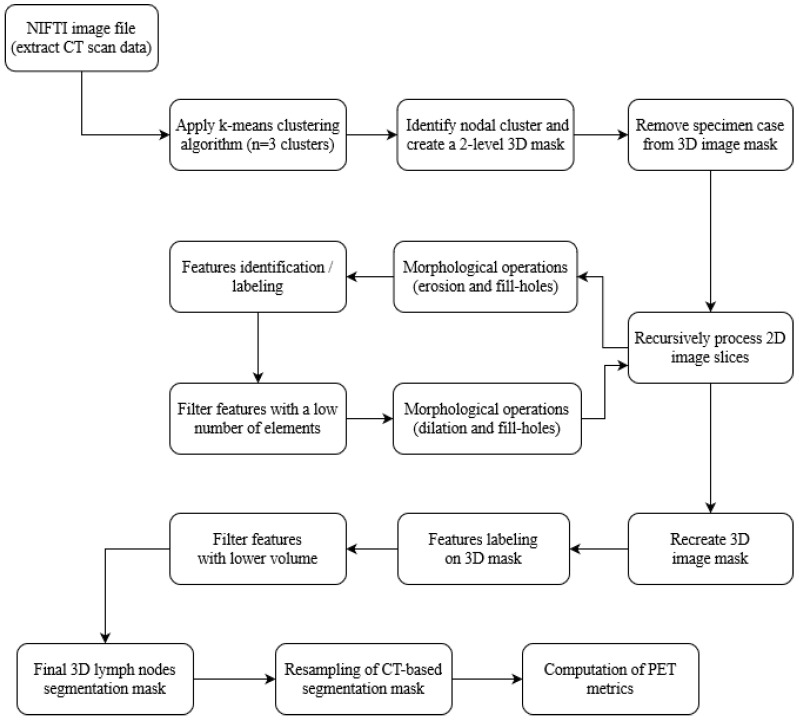
Model schema for the automatic CT-based nodal segmentation and PET semi-quantification of ^68^Ga-PSMA-11 PET/CT specimen images.

The complete model schema for the automatic nodal segmentation of the ^68^Ga-PSMA-11 PET/CT specimen images is presented in Figure 3.

**Figure 4:**
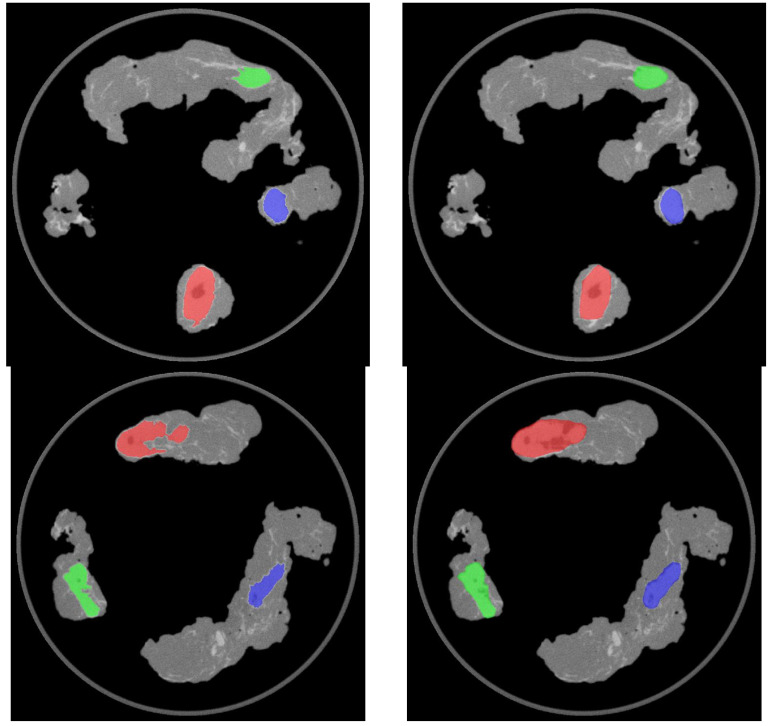
Axial images showing the outcome of the automatic (**left**) and manual (**right**) segmentation of the ^68^Ga-PSMA-11 PET/CT nodal specimen images. **Top row**: left pelvic lymph nodes. **Bottom row**: right pelvic lymph nodes.

The correspondence between the machine-learning automatic segmentation and the manual segmentation (see example in Figure 4) was evaluated using the following metrics: accuracy, precision, recall, Dice coefficient (F1 Score), and Jaccard index. 

The overall segmentation accuracy of both left and right pelvic lymph nodes was 99.7%; however, as expected, this parameter is overestimated due to the class imbalance deriving from the disparity between the target volume (i.e., lymph nodal structures) and the overall PET/CT field of view. 

The weighted precision of the automatic segmentation was ≥97% (range: 95–100%): indeed, the voxels segmented by the machine learning model correctly belonged to lymph nodal structures, and a low number of false positives was registered. On the other hand, the recall weighted average was 81% (range: 78–84%) in the first three specimens, but only 68% (range 64–72%) in the other three specimens. The Dice coefficient and Jaccard index also showed a comparable trend with weighted averages of 88% (range: 87–90%) vs. 80% (range: 78–84%) and 79% (range: 77–82%) vs. 67% (range: 64–72%), respectively. A detailed overview of the values of all the segmentation metrics for each specimen is presented in Table 1 and Table 2.

Finally, the segmentation masks were applied to the PET images to compute semi-quantitative metrics of tracer distribution such as the maximum standardized uptake value (SUVmax) and the target-to-background ratio (TBR). The SUVmax, TBR values and histopathology data of the nodal specimens are reported in Table 3. To perform this task, the CT-based segmentation masks were resampled to match the PET image specifications using the SITK library, while accounting for differences in size (1024 × 1024 × 512 vs. 252 × 252 × 152), spacing (0.1 vs. 0.4) and origin coordinates between the two images. The resampling was performed using the SITK NearestNeighbor interpolation method to avoid introducing new labels into the resampled image. The SUVmax values of the scanned lymph nodes ranged between 5.9–9.0 and 4.4–7.8 for the left and right pelvic lymph nodes, respectively. The manual and ML-based segmentation approaches yielded comparable SUVmax values since both strategies correctly segmented the area with the highest tracer uptake (Supplemental Figure S1). The TBR values ranged between 3.5–5.3 and 2.4–4.2 for the left and right pelvic lymph nodes, respectively (background SUVmax of left [1.7] and right [1.8] pelvic lymph nodes measured on non-target regions). No pathological lymph nodes were found at histopathology among the scanned specimens.

## 4. Discussion

This study represents the first described experience with machine learning (ML)-based automatic segmentation of intraoperative ^68^Ga-PSMA-11 PET/CT specimen images. 

The recent introduction of a new mobile PET/CT specimen scanner (AURA 10 specimen PET/CT imager, XEOS Medical NV, Belgium) has enabled surgeons and nuclear medicine specialists to leverage the diagnostic capabilities of PET/CT molecular imaging directly in the operating room for the assessment of the resection margins status and nodal involvement. Compared to the time-consuming frozen section technique, intraoperative molecular imaging can provide PET/CT data within 15 min from excision, thus reducing the operational time. Promising preliminary results have been described in the evaluation of PSMA-expressing prostate cancer [20], but also in breast cancer [17], head and neck cancer [18], pancreatic adenocarcinoma [19] and neuroendocrine tumors [20]. However, in order to accurately analyze the radiotracer uptake in PET/CT specimen images (i.e., lymph nodes) and obtain semiquantitative measurements that can improve the detection of disease localizations, accurate volumetric segmentation of 3D tomographic images is necessary. Contrary to the time-consuming manual segmentation, automatic 3D segmentation could contribute to streamline image analysis and ensure a higher degree of standardization and reproducibility in the evaluation of the radiotracer uptake. Therefore, the aim of this study was to test the feasibility of using machine learning to perform automatic nodal segmentation of intraoperative ^68^Ga-PSMA-11 PET/CT specimen images obtained from a high-risk prostate cancer patient undergoing robot-assisted radical prostatectomy (RARP) and pelvic lymph node dissection (PLND).

Compared to the manual segmentation, the newly developed ML-based automatic segmentation model showed an overall good performance in terms of accuracy, precision, recall, Dice coefficient and Jaccard index. The segmentation accuracy of both PET/CT specimen images was >99%, but this value was overestimated due to the class imbalance resulting from the limited volume of the target structures compared to the total scanned volume. A more realistic estimation of the segmentation accuracy is therefore provided by the other segmentation metrics. The 3D volumes segmented by the ML model showed a very good correspondence with lymph nodal structures, resulting in a few false positive results and weighted precision scores ≥ 97%. The morphological erosion of wrongly connected components in the segmentation masks and the subsequent implementation of noise removal strategies based on 2D/3D-features filtering contributed to enhance the model precision. 

The model correctly segmented 80% of the total volume of the resected lymph nodes in the first specimen PET/CT image, while a lower performance was recorded in the other specimens, possibly due to their more irregular structure and heterogeneous density leading to a higher rate of false negative results and a lower weighted average recall (68%). Although morphological operations, such as hole-filling, partially mitigated this problem by expanding the segmentation to also include regions with lower density values (e.g., lymph node hilum), further optimizations in shape recognition may contribute to improve the recall scores.

Overall, considering the Dice coefficients and Jaccard index weighted averages (80–88% and 67–79%), a similar approach could represent a viable starting point to facilitate the intraoperative PET/CT specimen image analysis and support the localization of nodal disease in prostate cancer patients directly in the operating room.

Promising evidence from previous studies has demonstrated a good correspondence between the lymph nodal PSMA uptake at intraoperative PET/CT imaging and the metastatic tumor involvement at histopathological analysis [20,21]. These findings are in agreement with the results of a preliminary case series analysis of three high-risk prostate cancer patients undergoing RARP and PLND with the use of the intraoperative PET/CT specimen imager performed at our center [34]. Specifically, in one of these patients, a suspicious left obturator nodal metastasis was detected during staging with whole-body ^68^Ga-PSMA-11 PET/CT. At intraoperative PET/CT specimen imaging, a higher degree of ^68^Ga-PSMA-11 uptake was also shown in a left obturator node compared with other resected pelvic lymph nodes. Accordingly, only one lymph nodal metastasis was confirmed at histopathological analysis within the left obturator nodes, while no other metastases were identified in the reaming nodal specimens. The other two patients of the case series showed no uptake or a more diffuse and milder uptake in the resected nodal specimens at intraoperative PET/CT imaging, and no lymph nodal metastases were identified at histopathology. Given the correspondence between tracer uptake and metastatic involvement, the use of semi-quantitative metrics (e.g., SUVmax) can facilitate specimen PET/CT interpretation by providing a more objective assessment of the nodal tracer uptake. Considering the variability in background uptake and timing of intraoperative PET/CT acquisition, the use of a target to background ratio (TBR)—calculated as the ratio between the SUVmax of the scanned lymph nodes and the background SUVmax—could prove to be more reliable than the absolute SUVmax. The TBR values of the scanned specimens ranged between 3.5–5.3 and 2.4–4.2 for the left and right pelvic lymph nodes, respectively. As reported in the literature, the TBR of metastatic lymph nodes has been shown to be considerably higher (e.g., 13.6) compared to disease-free lymph nodes.

In this context, the machine learning-based automatic nodal segmentation model presented in this study could help in automating the evaluation of the TBR and thus the interpretation of the intraoperative PET/CT images.

Although the present study implemented a clustering model, multiple approaches have been utilized to perform automatic segmentation of whole-body PET/CT images, including thresholding, atlas-based methods, machine learning and deep learning methods.

In a previous study on prostate cancer by Xu et al., convolutional neural networks (CNNs) with weighted batch-wise dice loss (wDice) were trained on 525 whole-body ^18^F-DCFPyL PET/CT scans to perform fully automated detection and segmentation of metastatic lesions [27]. On average, the model achieved an 80% detection rate for all testing lesions, and a 93% detection rate for lesions with a maximum standardized uptake value (SUVmax) higher than 5.0. The average median Dice similarity coefficient (DSC) for all lesions was 0.51, while for lesions with SUVmax > 5.0 was 0.60. The accuracy of segmentation proved to be heavily influenced by factors such as lesion intensity, size, and location. CNNs have also been successfully applied to whole-body ^18^F-FDG PET/CT images. Jemaa et al. developed a novel approach based on 2D and 3D CNNs to effectively identify and segment tumor localizations in 3664 ^18^F-FDG PET/CT scans of patients with non-Hodgkin’s lymphoma (NHL) and advanced non-small cell lung cancer (NSCLC) [35]. CNNs were trained on 2266 scans from diffuse large B cell lymphoma (DLBCL) patients and tested on 1124 follicular lymphoma (FL) and 274 NSCLC PET/CT scans. The model showed a mean 3D Dice score of 88.6% for NHL scans (FL test dataset) and a voxel-level sensitivity of 92.6% and 93.0% for the FL and NSCLC test sets, respectively. This approach allowed for the rapid assessment of the FDG-avid tumor burden: the estimated total metabolic tumor volume (MTV) and SUVmax showed a high Spearman’s correlation of 0.97 and 0.96, respectively, when compared to manual segmentation-based metrics. The improved availability of tools for automatic segmentation and assessment of the metabolic tumor burden in patients with solid tumors or lymphomas could inform clinical risk stratification and potentially guide patient management in the future [36]. Nevertheless, there are still significant technical challenges to overcome in order to perform automated image analysis, such as taking into account healthy tissues with high-metabolic activity, the relatively small volume of FDG-avid tumors compared to the total FDG-positive regions, the tumors heterogeneity in FDG uptake, and the variability of imaging acquisition protocols. 

Further promising results for the CNN-based automatic segmentation of primary functional tumor volumes in hybrid imaging have also been reported by Intsen et al., who investigated the application of CNNs to a multicenter dataset composed of ^18^F-FDG PET/CT scans of 232 cervical cancer patients [37]. Compared to a semi-automated approach based on the Fuzzy locally Adaptive Bayesian (FLAB) algorithm, the model built with a U-Net architecture achieved a good Dice similarity coefficient (DSC) (0.80 ± 0.03), recall (0.90 ± 0.05) and precision (0.75 ± 0.05), outperforming a fixed SUVmax threshold model (DSC 0.33 ± 0.15, recall 0.52 ± 0.17, precision 0.30 ± 0.16). 

Since deep learning models have demonstrated a superior performance for the automatic segmentation of medical images compared to other strategies [38], they could represent a promising approach also for the segmentation of intraoperative PET/CT imaging. However, their implementation in this setting is limited by the need for a large, labelled training dataset. Fortunately, in this use-case, the simplified anatomical context of specimen images allowed to achieve good performances using a clustering model. 

Finally, in the context of whole-body PET/CT image analysis, the availability of an automatic segmentation strategy could facilitate the deployment of an automated radiomics pipeline aimed at integrating clinically relevant image-features into models predictive of outcome or for assessing changes in tumors before, during, and after treatment [39,40]. Similarly, future research perspectives in intraoperative PET/CT imaging could include the correlation of features extracted from specimen molecular imaging with clinical data in order to improve the detection of disease localizations and the staging accuracy. In this context, it should be noted that the segmentation method can have a significant impact on the extracted radiomic features [41,42] and the automation of this step of the radiomics workflow could solve the issues of inter-/intra-operator segmentation variability, as well as the scalability of manual methods to handle large volumes of imaging data.

### Limitations

Despite the valuable insights gained from this study, it is essential to recognize the limitations inherent in the study design. Indeed, this pilot study was conducted on a limited number of specimen images and further testing on multiple PET/CT scans will be required to identify more edge-cases that could lead to misclassification errors. Additional tuning of the morphological operations and noise filtering process will also be needed to ensure a consistent performance. The evaluation of the impact of the morphological transformations on the accuracy of the automatic segmentation could not be performed since it would require a greater number of samples (representative of the different nodal anatomical variants). However, examples of the results of the processing steps are presented in Figure 2. Regarding PET semi-quantification, the SUVmax values reported in this study served only to demonstrate the application of the segmentation masks to the PET data; more detailed analyses involving other PET metrics will be needed to investigate the predictive value of molecular imaging. Although, in this study, the intraoperative PET/CT imaging results could not be used to guide the surgical treatment plan (which according to current guidelines was based on risk stratification), the presented ML-based segmentation approach could help to facilitate and standardize the collection of the data required for the validation of the intraoperative PET/CT technology. Cost-effectiveness aspects will also have to be evaluated after data regarding the clinical impact of this new technology become available. Finally, prostate cancer cells might also be present within the extra-nodal fatty tissue; this aspect should be taken into account for further improvements. Despite these limitations, the approach described in this study yielded promising results and the highlighted key-points could provide a basis for future improvements. Furthermore, this study made exclusive use of open-source libraries, thus facilitating its replicability. Finally, the know-how derived from this project could facilitate further research on automatic segmentation in more complex settings (e.g., whole-body ^68^Ga-PSMA-11 PET/CT imaging), as well as the investigation of the predictive value of semi-quantitative specimen PET/CT metrics compared to histopathology results.

## 5. Conclusions

The newly developed machine learning-based approach for automatic lymph nodal segmentation of intraoperative ^68^Ga-PSMA-11 PET/CT specimen images showed promising results compared to the manual segmentation in terms of weighted average precision (97–99%), recall (68–81%), Dice coefficient (80–88%) and Jaccard index (67–79%). This approach could represent a good complement to the capabilities of the intraoperative PET/CT scanner for the assessment of nodal involvement in surgical specimens, facilitating the semi-quantitative analysis of PET/CT images directly in the operating room.

## Figures and Tables

**Table 1:** Performance of the automatic segmentation model in the left pelvic lymph nodes.

**Specimens**	**Precision**	**Recall**	**Dice Coefficient**	**Jaccard Index**
Left proximal external iliac	0.98	0.78	0.87	0.77
Left obturator (1)	0.98	0.84	0.90	0.82
Left obturator (2)	0.95	0.82	0.88	0.79
**Average metrics**	**Precision**	**Recall**	**Dice Coefficient**	**Jaccard Index**
Micro average	0.97	0.81	0.88	0.79
Macro average	0.97	0.81	0.88	0.79
Weighted average	0.97	0.81	0.88	0.79

**Table 2:** Performance of the automatic segmentation model in the right pelvic lymph nodes.

**Specimens**	**Precision**	**Recall**	**Dice Coefficient**	**Jaccard Index**
Right distal external iliac	0.99	0.72	0.83	0.71
Right proximal external iliac	0.99	0.64	0.78	0.64
Right obturator	1.00	0.72	0.84	0.72
**Average metrics**	**Precision**	**Recall**	**Dice Coefficient**	**Jaccard Index**
Micro average	0.99	0.68	0.81	0.67
Macro average	0.99	0.69	0.81	0.69
Weighted average	0.99	0.68	0.80	0.67

**Table 3:** Maximum standardized uptake value (SUVmax), target-to-background ratio (TBR) and histopathology of the pelvic nodal specimens scanned with the intraoperative PET/CT scanner.

**Left Pelvic** **Lymph Nodes**	**SUVmax**	**Target-to-Background** **Ratio (TBR)**	**Lymph Node** **Histopathology**
Left proximal external iliac	5.9	3.5	Negative
Left obturator (1)	6.2	3.7	Negative
Left obturator (2)	9.0	5.3	Negative
**Right pelvic** **lymph nodes**	**SUVmax**	**Target-to-background** **Ratio (TBR)**	**Lymph node** **histopathology**
Right distal external iliac	7.8	4.2	Negative
Right proximal external iliac	4.4	2.4	Negative
Right obturator	7.0	3.8	Negative

## Data Availability

No new data were created or analyzed in this study. Data sharing is not applicable to this article.

## Supplementary Materials

The following supporting information can be downloaded at: https://www.mdpi.com/article/10.3390/diagnostics13183013/s1.
