# Supplementary material for: Machine Learning CT-Based Automatic Nodal Segmentation and PET Semi-Quantification of Intraoperative 68Ga-PSMA-11 PET/CT Images in High-Risk Prostate Cancer: A Pilot Study
Source: Diagnostics (Basel). 2023 Sep 21;13(18):3013. doi: 10.3390/diagnostics13183013 (PMC10529304; doi:10.3390/diagnostics13183013)
Supplement: Supplementary file 1 [file diagnostics-13-03013-s001.zip › diagnostics-2506043-supplementary.pdf]

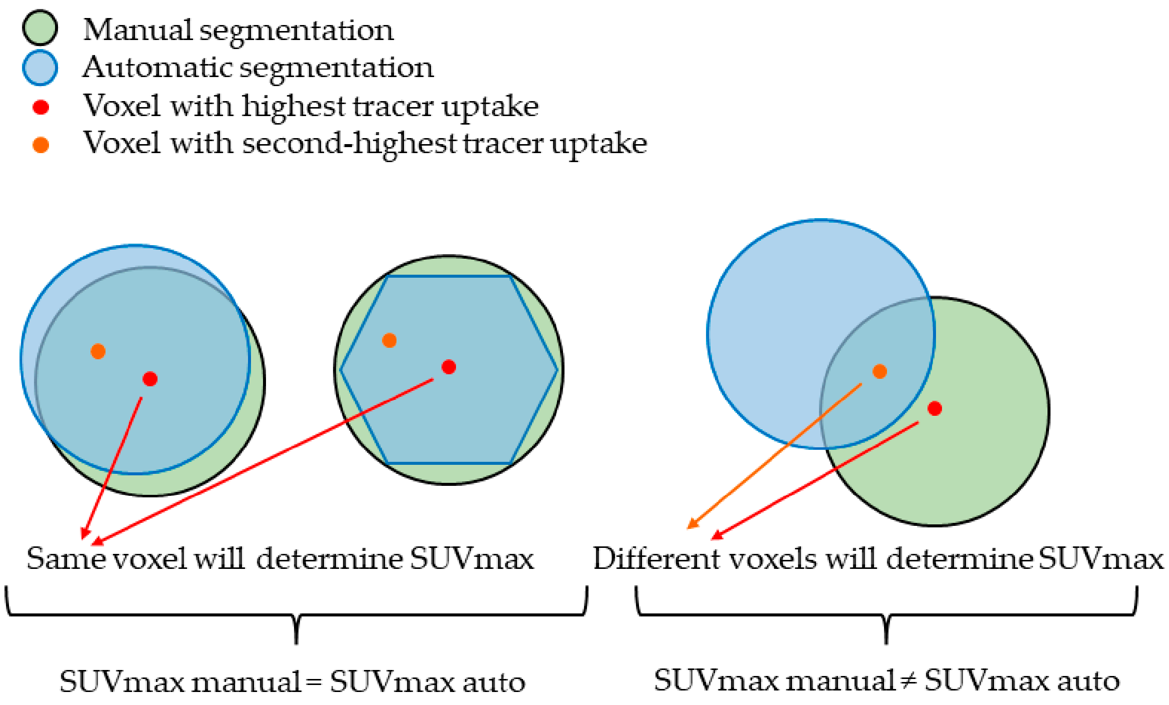
Manual segmentation
Automatic segmentation
Voxel with highest tracer uptake
Voxel with second-highest tracer uptake
Same voxel will determine SUVmax
Different voxels will determine SUVmax
SUVmax manual = SUVmax auto
SUVmax manual ≠ SUVmax auto
